# Supplementary material for: Gut microbes mediate the synergistic effects of dietary cholesterol and saturated fat in driving fibrosing MASH
Source: bioRxiv. 2025 Jul 21:2025.07.16.665145. Preprint. [Version 1] doi: 10.1101/2025.07.16.665145 (PMC12330582; doi:10.1101/2025.07.16.665145)
Supplement: Supplement 4 [file NIHPP2025.07.16.665145v1-supplement-4.pdf]

bioRxiv preprint doi: https://doi.org/10.1101/2025.07.16.665145; this version posted July 21, 2025. The copyright holder for this preprint (which was not certified by peer review) is the author/funder, who has granted bioRxiv a license to display the preprint in perpetuity. It is made available under aCC-BY 4.0 International license.

Figure S1. GF and SPF mice exhibit comparable caloric intake and adiposity, but disparate liver lipid and fibrosis profiles.

A) Caloric intake attributed to water, food, or combined during the duration of the study. Data represent means ± SEM, analyzed via 4-way RM ANOVA (factors: Cholesterol, Fat, Microbes, Time) followed by 3-way ANOVA (factors: Cholesterol, Fat, Time) within SPF and GF groups and Tukey's

bioRxiv preprint doi: https://doi.org/10.1101/2025.07.16.665145; this version posted July 21, 2025. The copyright holder for this preprint (which was not certified by peer review) is the author/funder, who has granted bioRxiv a license to display the preprint in perpetuity. It is made available under aCC-BY 4.0 International license.

multiple comparisons within timepoint. **P*<0.05. Asterisk color represents which group is significantly different from LF. **B,C)** Liver mass (B) and total white adipose tissue (WAT) mass (C) as a percent of body mass. Data represent means ± SEM analyzed via 3-way ANOVA. Comparisons made between SPF Week 8 vs. SPF Week 24 (factors: Cholesterol, Fat, Time, left panels) or between SPF Week 24 vs. GF Week 24 (factors: Cholesterol, Fat, Microbes, right panels), followed by Tukey's multiple comparisons within timepoint. Bars with the same letter are not significantly different (*P*>0.05). **D)** Representative Oil Red O-stained liver sections; scale bar=200µm. **E)** Quantification of percent area stained red (indicating neutral lipids). **F)** Representative Masson's Trichrome-stained liver sections; scale bar=200µm. Inset images (400x) correspond to boxed area in 100x images. **G)** Quantification of percent area stained blue (indicating collagen). Data represent means ± SEM, analyzed via 3-way ANOVA. Comparisons made between SPF Week 8 and SPF Week 24 (factors: Cholesterol, Fat, Time) or between SPF Week 24 and GF Week 24 (factors: Cholesterol, Fat, Microbes), followed by Tukey's multiple comparisons. Bars with the same letter are not significantly different (*P*>0.05).

Figure S2. Dietary cholesterol and saturated fat differentially impact fecal and cecal microbiota diversity and composition over time in SPF mice.

A,B) Chao1 (top left), Fisher's alpha (top right), Shannon index (bottom left), and Simpson index (bottom right) α-diversity indices of fecal (A) and cecal (B) microbiota in SPF mice throughout the study (A). Data represent means ± SEM, analyzed via 3-way ANOVA (Factors: Cholesterol, Fat, Time) followed by Tukey's multiple comparisons within timepoint. **P*<0.05, ***P*<0.01, ****P*<0.005. For fecal diversity indices, asterisk color represents significant differences from LF. **C,D)** Bray-Curtis β-diversity PCoA of fecal microbiota, analyzed via 3-factor ADONIS (Factors: Time, Cholesterol, Fat) (C) or 2-factor ADONIS (Factors: Cholesterol, Fat) (D). **E,F)** Unweighted (E) and Weighted (F) Unifrac β-diversity PCoA of cecal microbiota after 8 (left panels) and 24 (right panels) weeks on diet, analyzed via 2-factor ADONIS (Factors: Cholesterol, Fat). For all PCoA plots, dots represent individual mice, open circles represent centroids with lines connecting individual dots within a treatment group.

Figure S3. Distinct effects of dietary cholesterol and saturated fat on cecal microbiota composition at 8 and 24 weeks.

A-D) ASVs significantly enriched or depleted by dietary cholesterol (A,B) or saturated fat (C,D) via MaAsLin2 pairwise comparisons at 8 (A,C) and 24 (B,D) weeks on diet. Positive coefficients indicate enrichment; negative coefficients indicate depletion. Dot size reflects -log(P-value). Taxonomic lables: "f__": family-level annotation; "o__" order-level annotation; "c__" class-level.

bioRxiv preprint doi: https://doi.org/10.1101/2025.07.16.665145; this version posted July 21, 2025. The copyright holder for this preprint (which was not certified by peer review) is the author/funder, who has granted bioRxiv a license to display the preprint in perpetuity. It is made available under aCC-BY 4.0 International license.

Figure S4. Fecal BA profiles are altered in response to dietary cholesterol and saturated fat.

Heat map of fecal BA after 8 or 24 weeks in SPF mice, represented as Z-score of normalized peak area within each BA. BA with an asterisk(*) yielded quantitative values as shown in Figure 4G-L. Tα/β-MCA: Tauroα/β-muricholic acid; TCA: Taurocholic acid; GCA: Glycocholic acid; TCDCA: Taurochenodeoxycholic acid; GCDCA: Glycochenodeoxycholic acid; CA: Cholic acid; CDCA: Chenodeoxycholic acid; α/β/γ/ω-MCA: α/β/γ/ω-Muricholic acid; 3-epiCA: 3-epicholic acid; TDCA: Taurodeoxycholic acid; TUDCA: Tauroursodeoxycholic acid; THDCA: Taurohyodeoxycholic acid; TLCA: Taurolithocholic acid; GDCA: Glycodeoxycholic acid; GUDCA: Glycoursodeoxycholic acid; GLCA: Glycolithocholic acid; GDCA: Glycodeoxycholic acid; UCA: Ursocholic acid; alloCA: Allocholic acid; LCA: Lithocholic acid; isoLCA: Isolithocholic acid; alloLCA: Allolithocholic acid; alloisoLCA: Alloisolithocholic acid; β-HDCA: β-hyodeoxycholic acid; DCA: Deoxycholic acid; UDCA: Ursodeoxycholic acid; HDCA: Hyodeoxycholic acid; 3-DCA: 3-deoxycholic acid; 3-oxoCA: 3-oxocholic acid; 3-oxo(C)DCA: 3-oxochenodeoxycholic acid or 3-oxodeoxycholic; 7-/6-oxoLCA: 7-/6-oxolithocholic acid; 12-oxoLCA: 12-oxolithocholic acid; 3-oxoLCA: 3-oxolithocholic acid; 12-oxoCDCA: 12-oxochenodeoxycholic acid; 7-12-dioxoLCA: 7-12-dioxolithocholic acid; isoDCA: isodeoxyholic acid; 7-oxoDCA: 7-oxodeoxycholic acid.

Figure S5: LPS and TBA are not primary mediators of *in vitro* HSC activation induced by cecal homogenates.

A,B) LPS (A) and TBA concentrations (B) in cecal homogenates prepared as shown in Figure 5A. **C-G)** Individual BA concentrations in cecal homogenates. Data represent means ± SEM, analyzed via 3-way ANOVA (factors: Cholesterol, Fat, Microbes) followed by Tukey's multiple comparisons within timepoint. Bars with the same letter are not significantly different ($P>0.05$). α/β/ω-MCA: α/β/ω-Muricholic acid; CA: Cholic acid; GCA: Glycocholic acid; TCDCA: Taurochenodeoxycholic acid; CDCA: Chenodeoxycholic acid; LCA: Lithocholic acid; UDCA: Ursodeoxycholic acid.

bioRxiv preprint doi: https://doi.org/10.1101/2025.07.16.665145; this version posted July 21, 2025. The copyright holder for this preprint (which was not certified by peer review) is the author/funder, who has granted bioRxiv a license to display the preprint in perpetuity. It is made available under aCC-BY 4.0 International license.

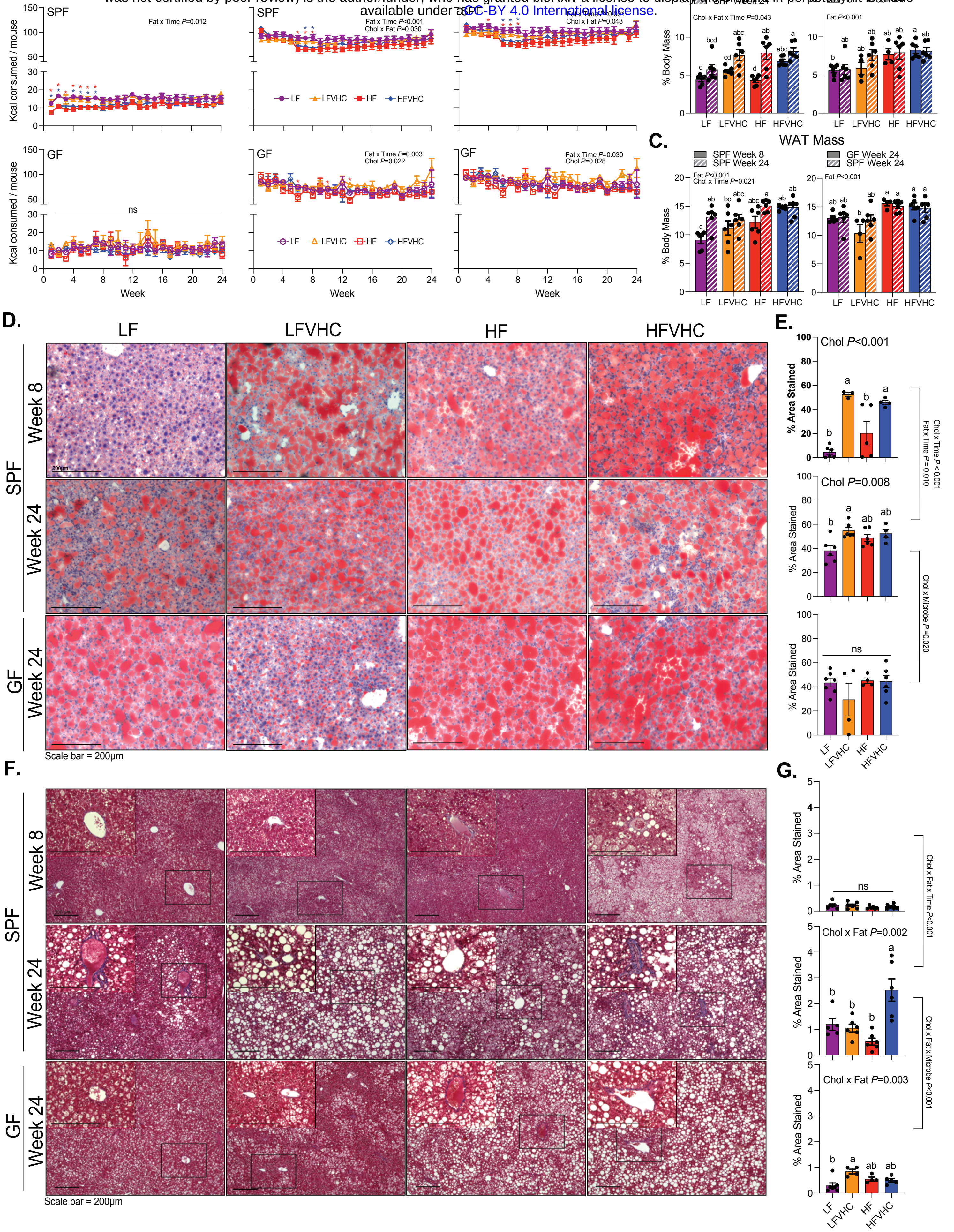

Supplementary Figure 1

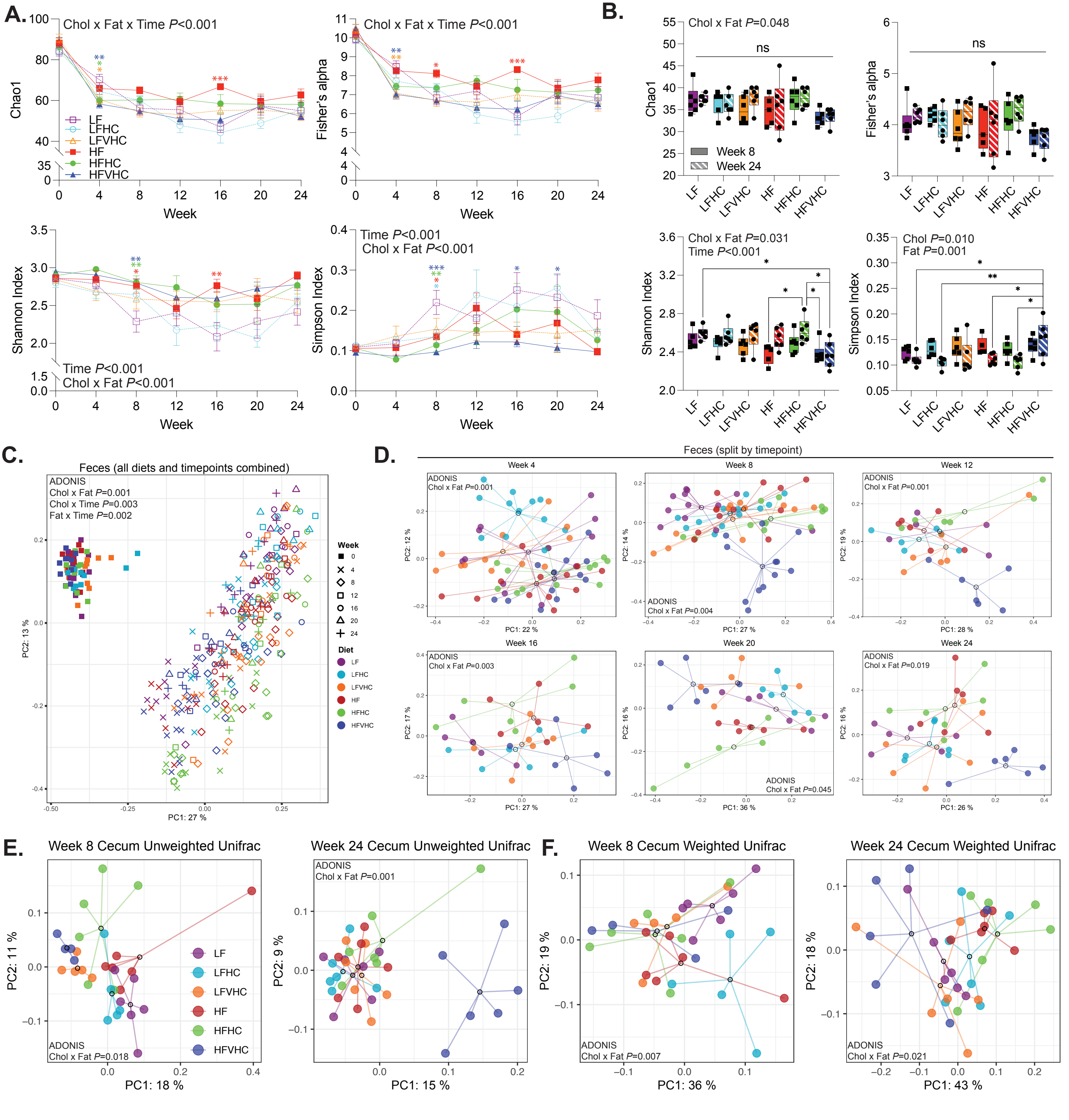

Supplementary Figure 2

bioRxiv preprint doi: https://doi.org/10.1101/2025.07.16.665145; this version posted July 21, 2025. The copyright holder for this preprint (which was not certified by peer review) is the author/funder, who has granted bioRxiv a license to display the preprint in perpetuity. It is made available under aCC-BY 4.0 International license.

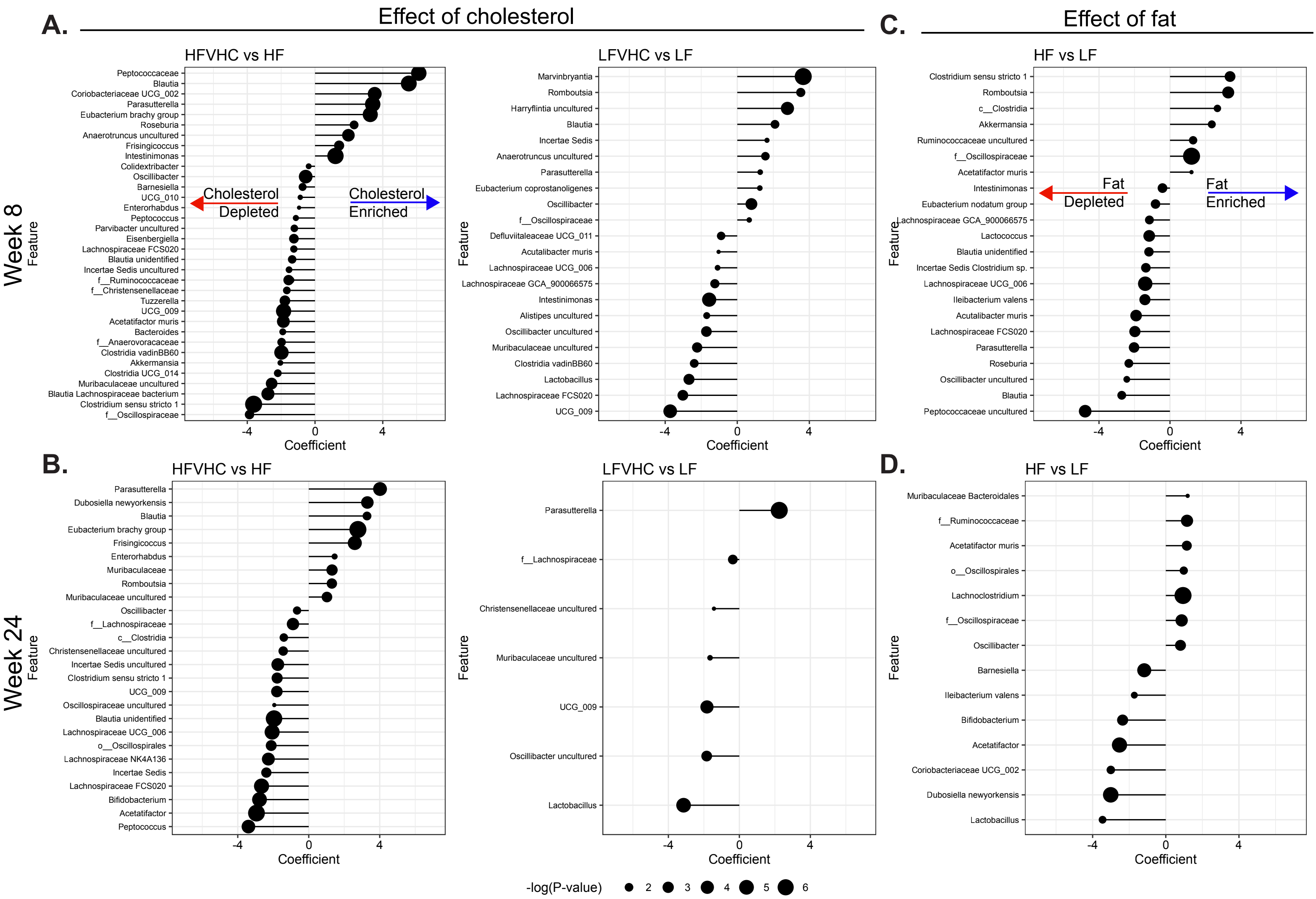

Supplementary Figure 3

bioRxiv preprint doi: https://doi.org/10.1101/2025.07.16.665145; this version posted July 21, 2025. The copyright holder for this preprint (which was not certified by peer review) is the author/funder, who has granted bioRxiv a license to display the preprint in perpetuity. It is made available under aCC-BY 4.0 International license.

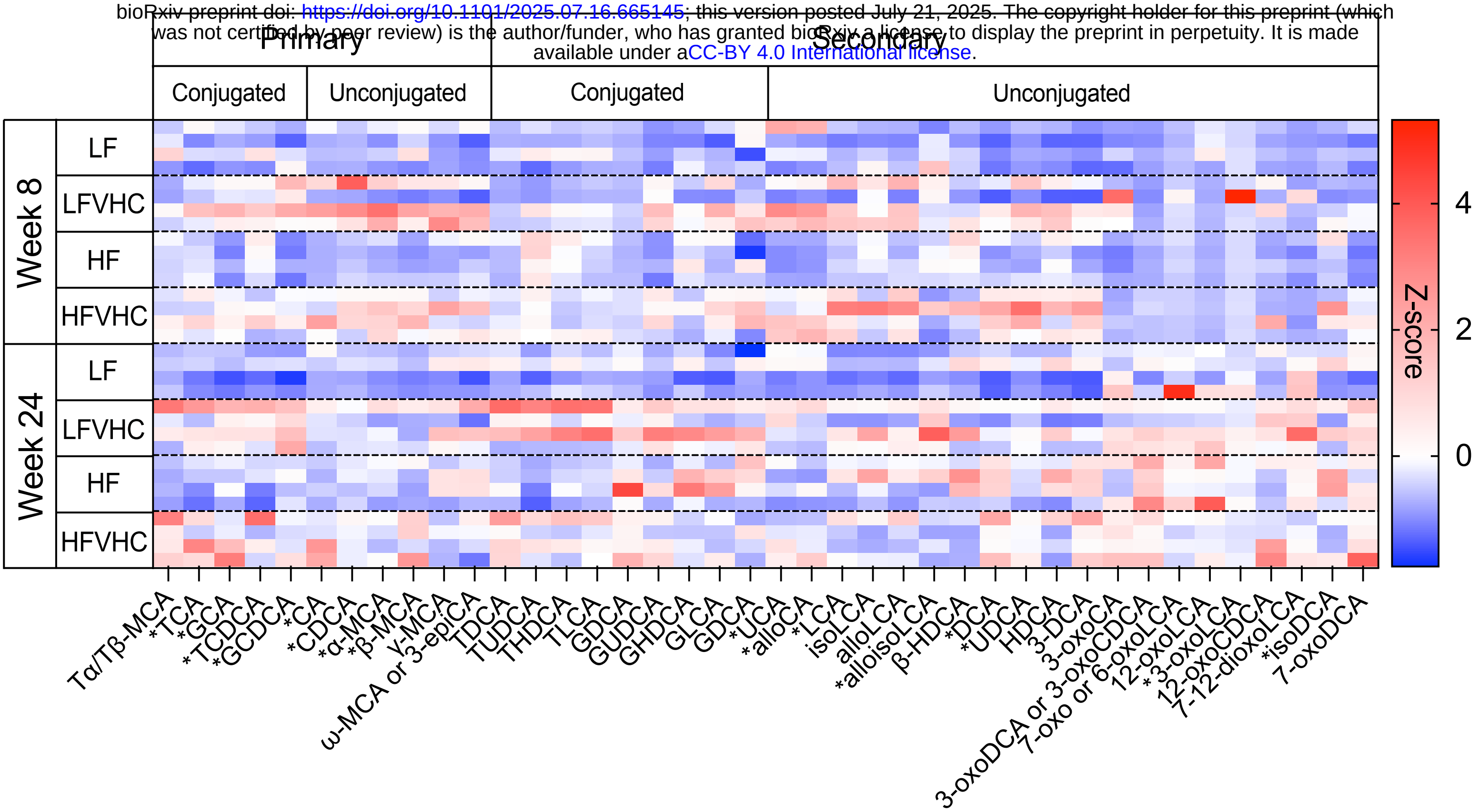

Supplementary Figure 4

bioRxiv preprint doi: https://doi.org/10.1101/2025.07.16.665145; this version posted July 21, 2025. The copyright holder for this preprint (which was not certified by peer review) is the author/funder, who has granted bioRxiv a license to display the preprint in perpetuity. It is made available under aCC-BY 4.0 International license.

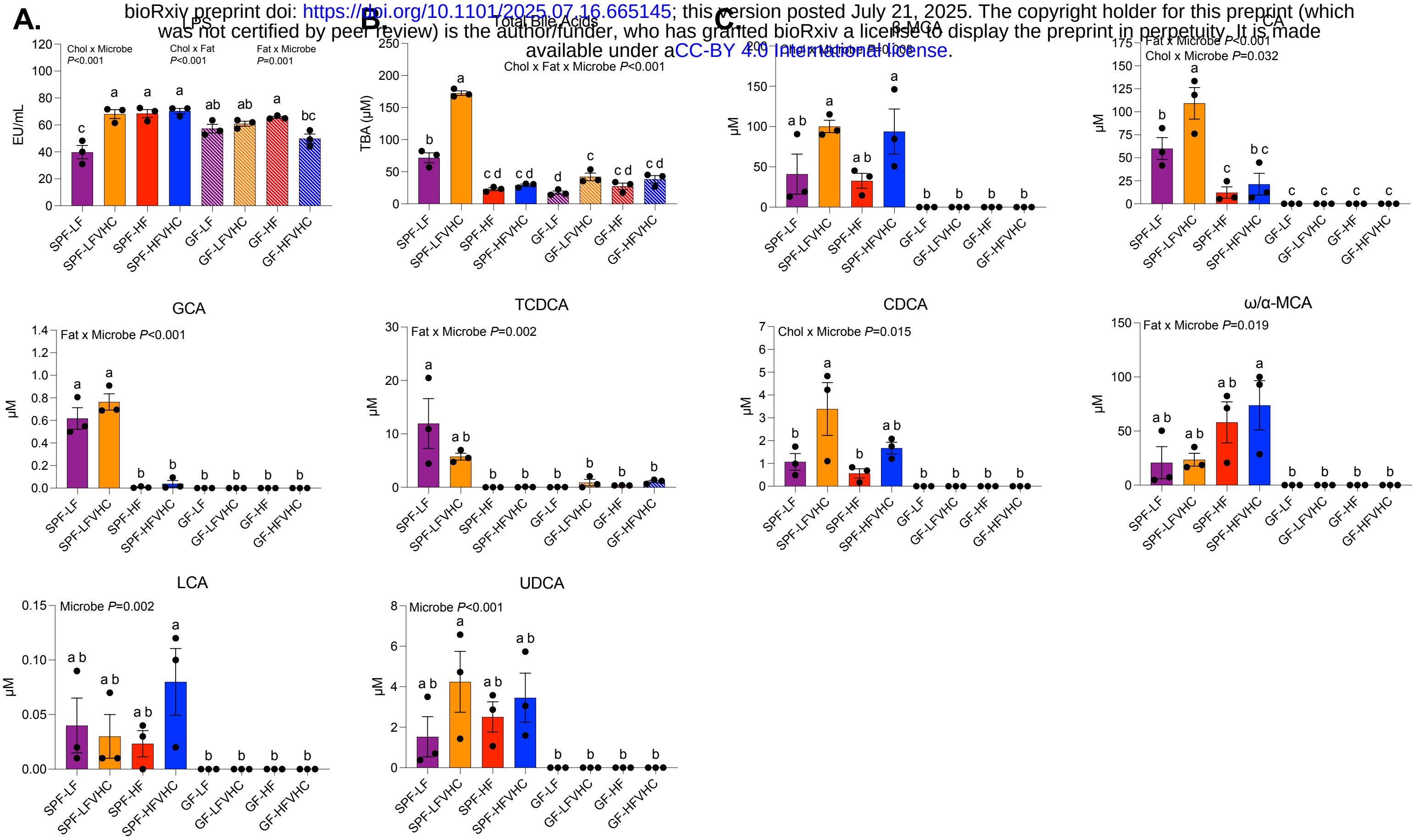

Supplementary Figure 5
